# A Gel Polymer Electrolyte Reinforced Membrane for Lithium-Ion Batteries via the Simultaneous-Irradiation of the Electron Beam

**DOI:** 10.3390/membranes11030219

**Published:** 2021-03-19

**Authors:** Jian Hou, In Kee Park, Woo Ju Cha, Chang Hyun Lee

**Affiliations:** Department of Energy Engineering, Dankook University, Cheonan 31116, Korea; houjimmy@naver.com (J.H.); inkee0149@gmail.com (I.K.P.); universe333@naver.com (W.J.C.)

**Keywords:** gel polymer electrolyte, reinforced membrane, electron beam, simultaneous irradiation

## Abstract

In this research, a series of innovative and stable cross-linked gel polymer reinforced membranes (GPRMs), were successfully prepared and investigated for application in lithium-ion batteries. Herein, a gel directly within the commercial polyethylene (PE) separator is supported via electron-beam simultaneous irradiation cross-linking of commercial liquid electrolyte and poly(ethylene glycol) methacrylate (PEGMA) oligomers. The physical and electrochemical properties of the GPRMs were characterized by SEM, TEM, mechanical durability, heating shrinkage, and ion conductivity, etc. The GPRMs demonstrated excellent mechanical durability and high ion conductivity compared with traditional PE membranes. Moreover, coin-typed cells were assembled and cycle performance was also studied compared with same-typed cells with commercial PE membrane and liquid electrolyte. As a result, the coin-typed cells using GPRMs also showed a relatively good efficiency on the 50th cycles at a high 1.0 C-rate. These GPRMs with excellent properties present a very promising material for utilization in high-performance lithium-ion batteries with improved safety and reliability.

## 1. Introduction

Lithium-ion batteries are one of the most promising and efficient high energy density systems for electrochemical energy storage. In recent years, due to the application of lithium batteries to general electronic devices and electric vehicles, the demand for more efficient and safe batteries has increased greatly [1,2,3,4,5]. For general secondary lithium-ion batteries, although its function as an ion conductive medium of rechargeable lithium battery is fulfilled by using an ion-conductive liquid electrolyte in which lithium salt is dissolved and a polyolefin separator to improve the transfer rate of lithium ions, unfortunately, dendrite growth that penetrates the membrane separating the anode and cathode can occur. The growth of dendrite causes an electrical short-circuit phenomenon, and the polyolefin-based separators have a risk of catching on fire or exploding due to their poor heat resistance, and liquid electrolytes may also leak [6,7]. In a lithium-ion battery, the separator plays the role of transferring lithium ions between the anode and cathode while at the same time functioning as a safety device that can block the battery circuit by blocking the pores of the support itself when there is a flow of overcurrent. Recently, as polyolefin separators have been pointed out as a major cause of lithium-ion battery explosions due to their weakness in mechanical strength and severe thermal deformation, a solution is required [8,9,10,11]. In addition, as the demand for high-capacity and high-durability batteries is increasing due to the recent increase in the demand for electric vehicles and energy storage systems, the above problem can be taken more seriously [12,13]. Accordingly, the development of a completely solid battery that does not use electrolytes is in the initial state of research, but due to the low fluidity of solid electrolytes, the ion mobility becomes slow, lowering the relative conductivity, and causes a problem of low efficiency [14,15]. Recently, there have been many studies on gel polymer electrolyte which was coated onto a porous separator under UV or plasma irradiation. However, these grafting prepolymers are very active and hence lead to storge problems [16,17,18]. Therefore, developing a highly durable electrolyte-separator with excellent ion conductivity at the level of a liquid electrolyte is an immediate problem of secondary lithium battery separators [19,20].

Compared to UV or plasma, an electron beam is a high-energy radiation emitted using a high-energy electron beam from an electron accelerator. When an electron beam with high energy is irradiated onto a material, the molecular structure of the material changes or a specific chemical reaction occurs, and harmful microorganisms are killed. In addition, it is possible to weld by focusing heat energy on a very small part in a vacuum atmosphere. Using these features, the electron beam can be used to develop new technologies that can be applied to various fields such as modification of polymers, welding, large-capacity power semiconductors, medical tools, food sterilization, wastewater treatment, and exhaust gas purification [21,22]. Radiation grafting technology is a technology that grafts other polymers by using radiation, which has a large amount of energy and the ability to penetrate substances. Graft technology can be used by several methods including UV radiation, plasma treatment, decomposition of chemical initiator, and electron beam irradiation. Among these methods, electron beam irradiation has the advantage of grafting polymers to achieve reaction on the outer surface and inside and does not require any initiators. Due to these advantages, the electron beam irradiation grafting technology has been used effectively to manufacture functional materials such as cation exchange membranes, battery separators, and adsorbents for harmful substances [23,24,25,26]. The radiation grafting method consists of the step of irradiating the desired polymer to generate radicals in the main and side chains of the polymer and the step of mixing the polymer in which the radical is formed with other monomers to form a graft polymer. When these steps are done separately, it is referred to as the pre-irradiation method and when the polymer and monomers mixture is carried out, it is called the simultaneous-irradiation method [27,28,29]. In our previous studies, novel double-layered cation exchange membranes were prepared by simultaneous electron beam irradiation and were successfully applied to the saline water electrolysis (SWE) single cells [30,31].

The objective of this work is to propose a facile synthesis and fabrication method of an innovative gel polymer electrolyte reinforced membrane using the electron-beam simultaneous irradiation technology. Via the simultaneous irradiation cross-linking of PEGMA oligomer in the presence of ionic liquid electrolyte, a three-dimensional cross-linked structure was formed within the commercial PE support membranes. This unique structure endows the gel polymer electrolyte reinforced membrane with excellent performance such as excellent mechanical durability, high ionic conductivity, and high stability against exposure to external air and moisture, etc.

## 2. Materials and Methods

### 2.1. Materials

Poly(ethylene glycol) methacrylate (PEGMA, 500 Da) was purchased from Sigma-Aldrich (Seoul, Korea), and a commercial liquid electrolyte used was LiPF_6_ (1.0 M) with ethylene carbonate (EC)/ethyl-methyl carbonate (EMC) (1:2 vol) supplied from Panax Etec (Seoul, Korea). A commercial polyethylene (PE) battery separator (15 µm in thickness) was kindly supplied by Samsung Advanced Institute of Technology (SAIT, Suwon, Korea). Aluminum packs were purchased from Wowpack (Seoul, Korea) and used to shield the outside air during the electron beam irradiation. All chemicals were used as received.

### 2.2. Membrane Fabrication

**Figure 1:**
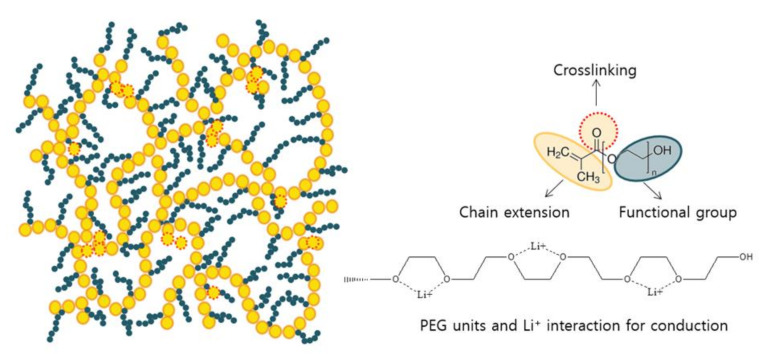
The schematic diagram of the gel polymer electrolyte reinforced membrane (GPERM) manufacturing process.

To fabricate the gel polymer electrolyte membrane, a radiation cross-linking method by electron beam was used. First, a certain amount of PEGMA was dissolved and stirred in the commercial liquid electrolyte to become a homogenous solution, wherein the target concentration of PEGMA was fixed at 1, 10, 20, 40, and 60 wt.%. Afterward, a commercial PE separator as porous support was soaked in the solution and sonicated for 1 h to form a composite film. After swelling, the excess mixture on the surface was removed by wiping lightly using filter paper. Subsequently, the swollen film was placed into an aluminum pack and sealed. All the processes from PEGMA dissolution to aluminum packaging were conducted in an Ar-filled glove box (SK-G800, Three-Shine INC., (Daejeon, Korea) where oxygen and H_2_O contents were controlled below 1.0 ppm. Since aluminum is known as an excellent barrier material to the passage of gases including water vapor and oxygen [32], the use of the aluminum pack was effective in preventing the penetration of moisture and oxygen during the electron beam irradiation. The electron beam under the conditions of 50 KGy, 1.4 MeV, 1 mA was simultaneously irradiated for 30 s, using a large electron beam accelerator (Normal-conducting RF linac electron accelerator, Korea). Finally, it was treated for 3 h in an oven at 55 °C to form the gel polymer electrolyte reinforced membrane (GPERM). Figure 1 is a schematic diagram of the manufacturing process of the gel polymer electrolyte reinforced membrane. Here, the PEG oxy-ethylene (–CH_2_CH_2_O–) unit is known to make strong ion-dipole interaction with Li^+^ ions. The ion-dipole interaction is so strong that Li^+^ ions are not extracted even in the carbonate-based liquid electrolyte phase [33,34,35,36,37]. Meanwhile, the PEG units are helpful in transporting Li^+^ ions for conduction owing their high flexibility. The CH_2_=CH_3_ in PEGMA is designed to form main chains marked in yellow circle, while the carbonyl group marked in red dot line is used for chemical crosslinking via electron beam treatment [38]. The side chain composed of the oxyethylene unit is highlighted in cyan.

### 2.3. Characterizations

The surface morphology of the commercial PE separator and the gel polymer electrolyte reinforced membranes (GPERM) were observed by the mean of field emission scanning electron microscope (FE-SEM, Model JES-6701F, JEOL, (Tokyo, Japan). All the surfaces of the samples were coated with a thin Pt layer before SEM measurements. Moreover, the microstructure of the GPERs was studied using a transmission electron microscope (EF-TEM, Model LIBRA 120, Carl Zeiss, (Stuttgart, Germany) operated at an acceleration voltage of 120 kV. Before the measurement, it undergoes three pretreatment steps of fixation, drying, and embedding, and the fixed sample was finely cut using an Ultramicrotome (Model EM UC7, Leica, (Wetzlar, Germany) and placed on a grid for TEM analysis.

**Figure 2:**
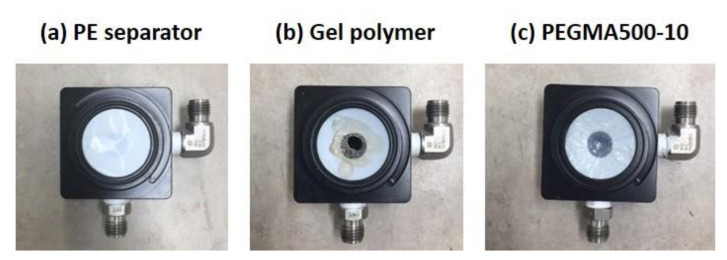
Structure of the pressure-loaded blister test cell.

The thermal shrinkage rate is commonly used as one of the most important characteristics to evaluate thermal stability. Hence, the thermal shrinkage rate of a membrane at the evaluated temperature was investigated by treating the sample in a vacuum oven at different temperatures (0 °C to 140 °C) for 1 h. The thermal shrinkage rate was determined by measuring the dimensional change before and after treating the sample at different temperatures for 1 h and the shrinkage rate was calculated by the equation:

(1) $$Thermal\;shrinkage\;rate\left(\%\right)=\;\left(\frac{A_{0}-A_{1}}{A_{0}}\right)\times 100\%$$

where the *A*_0_ and *A*_1_ are the area of the membrane before and after treatment [39]. In addition, a pressure-loaded blister test was carried out to analyze the mechanical durability of the commercial PE separator, pure gel polymer, and gel polymer electrolyte reinforced membrane (GPERM). Figure 2 shows the structure of the pressure-loaded blister test cell and a picture of it after the test. Air is injected by fastening the membrane to be tested for durability inside as shown in the figure. The analysis is carried out by measuring the time until a 15% loss of the initially applied pressure occurs by momentarily applying specific pressure to only one direction after loading the same gas pressure on both sides of the measurement sample [40,41]. It is possible to evaluate the durability of the membranes in a relatively short time using this method. For the measurement conditions, feed gas air pressure of 0.5 bar with 30% RH relative humidity was applied and the experiment was repeated every 1 s at a temperature of 65 °C.

The ionic conductivity of membranes was determined from the bulk resistance value (*R*). and a 2-probe AC complex impedance spectroscopy method using Potentiostat (Model VSP, BioLogic, (Seyssinet-Pariset, France) was used by setting an AC signal with an amplitude of ±20 mV in the frequency range of 1 MHz–500 µHz [42]. In addition, to verify the stability against exposure to external air and moisture, the ionic conductivity of the samples based on different storage times in the oven (25 °C) were also tested. The ionic conductivity was calculated from the bulk resistance value according to the following equation:

(2) $$\sigma =\frac{L}{R\times S}$$

where *R* is the bulk resistance, *L* is the thickness and *S* is the effective area of the membrane, respectively. The charge-discharge cycling stability is a common index in the electrochemical performance of lithium-ion batteries. Two electrodes were prepared prior to designing the coin cell batteries. The cathode was prepared by mixing the LiCoO_2_, PVDF binder and super P carbon (85:7.5:7.5 wt.%) in NMP solvent. The resulting mixture was coated with ~100 µm thickness onto aluminum foil (10 µm thick) using a Comma Coater. The electrode was then dried and pressed with a compression rate of 30% (i.e., the thickness reduction of the electrode reached ~70% of its original sample after the pressing step). The anode was prepared by mixing the natural graphite, PVDF binder and super-P carbon (90:7:3 wt.%) in the NMP solvent. The mixture was coated with an approximatively 110 µm thickness on copper foil (10 µm thick), which was then completely dried. The resulting electrode was then pressed with a compression rate of 25%. The coin cell using gel polymer electrolyte reinforced membrane (16–21 µm thick) was assembled and evaluated and compared with the coin cell made by the commercial PE separator (15 µm thick) and liquid electrolyte. All the cells were measured over 50 cycles by 1.0 C-rate at room temperature using a charge/discharge life analyzer (PEBC050.1, PNE solution, (Suwon, Korea) [43,44].

## 3. Results and Discussion

**Figure 3:**
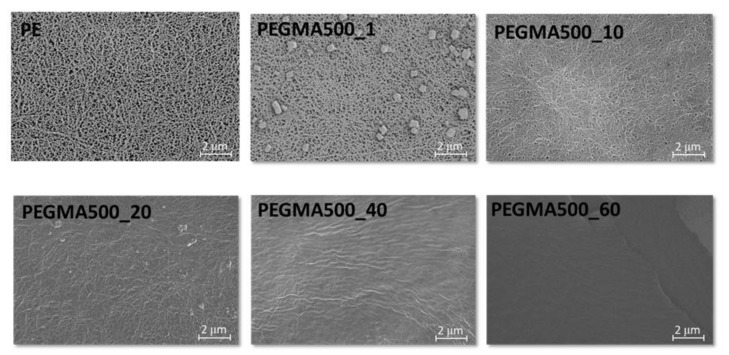
SEM images of the commercial polyethylene (PE) separator and GPERMs.

**Figure 4:**
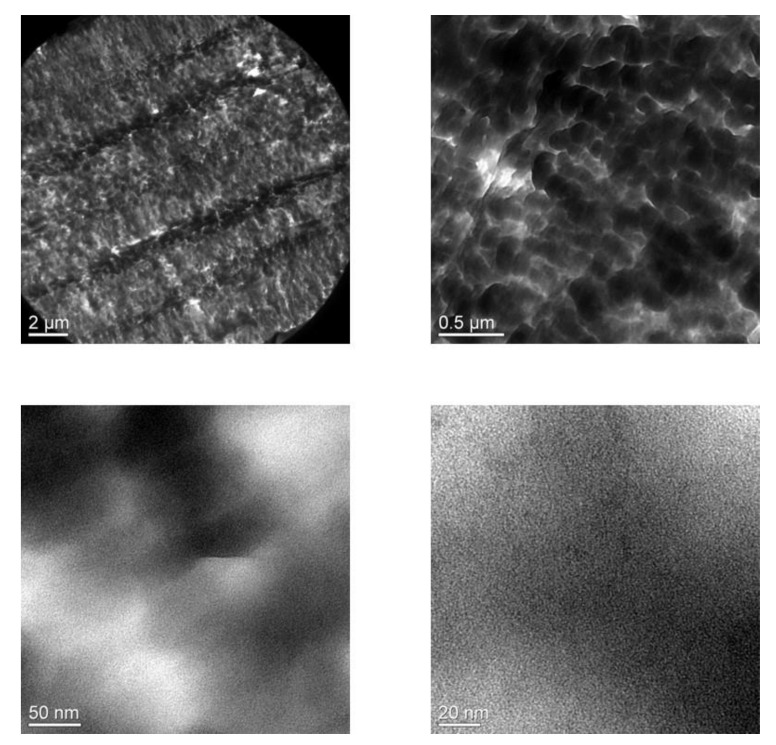
TEM images of the representative GPERM (PEGMA500_10).

Figure 3 shows the SEM morphology of the GPERMs according to the changes in the concentration of PEGMA. Compared with the morphology of the commercial PE support, when 1 wt.% of PEGMA was used, it can be seen that somewhat uneven gelation occurs inside and outside the PE support due to the low concentration. However, when 10 wt% or more of PEGMA is introduced, gelation begins to occur in the pore structure of the PE support, and when 40 wt% or more is introduced, it can be seen that gelation occurs not only in the pore structure of the PE support but there is excess gelation to cover the pores of the surface. This unique gelation method results in an increase in the thickness of the created gel polymer electrolyte reinforced membrane. Figure 4 shows the TEM morphology measured without a separate staining process for the gel polymer electrolyte reinforced membrane. Considering that a specimen with high electron density appears black in TEM analysis, the black color in each image indicates the distribution of lithium salt. As expected from the schematic diagram of the chemical structure network in Figure 1, the lithium salt was distributed in a dense form, and it was confirmed that it appeared in a pouch form. In addition, as seen from the high-magnification TEM image, it can be seen that the lithium salt inside each pouch is distributed in an even dispersion state. It was assessed that the evenly concentrated distribution of lithium salts inside the pouch would have a positive effect on ion conductivity.

**Figure 5:**
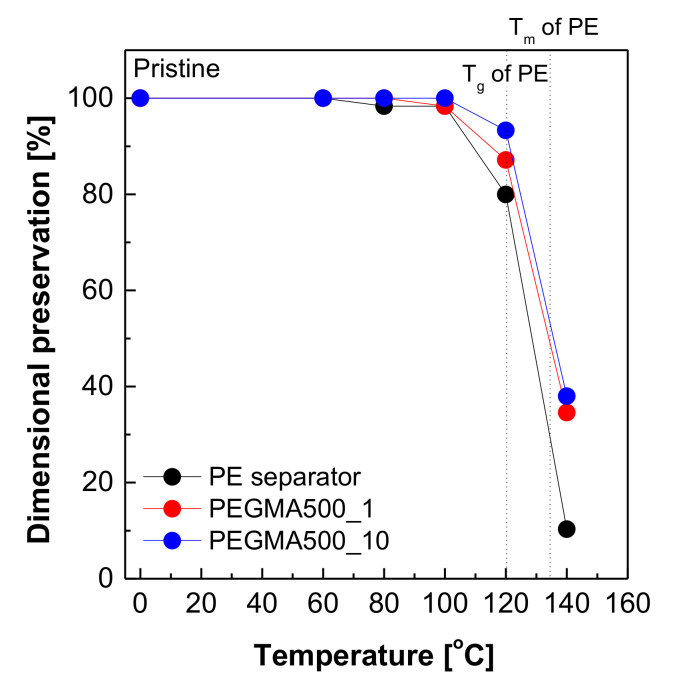
Thermal shrinkage rate of the commercial PE separator and GPERMs.

Figure 5 shows the change in the thermal shrinkage rate of the gel polymer electrolyte reinforced membrane. The commercial PE separator used as a support starts isotropic shrinkage from 80 °C near the glass transition temperature of PE polymer (~78 °C), as can be known from the intrinsic function of the separator, and up to 20% change in measurement occurs at 120 °C. On the other hand, for the gel polymer electrolyte reinforced membrane filled with gelatinized PEGMA in the pore structure, it begins to shrink from 100 °C, and for PEGMA500_10, it shows the lowest change in measurement (~7%) at 120 °C. This indicates that the moderate gel polymers can contribute to improving heat resistance when impregnated in a pore structure.

**Figure 6:**
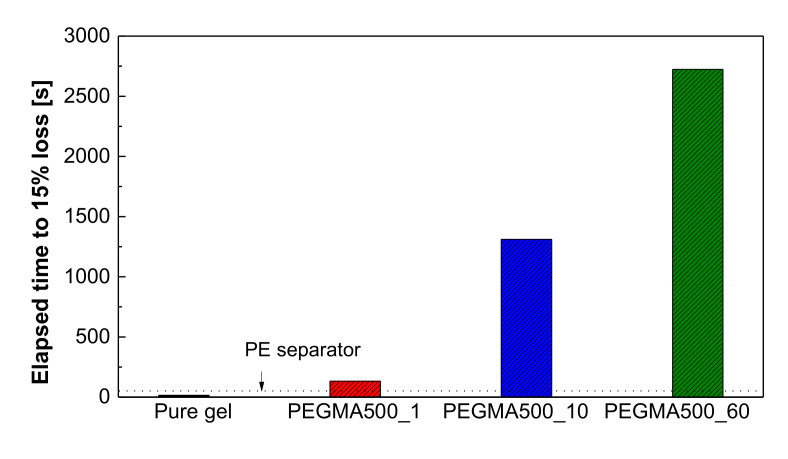
Durability analysis result according to the physical acceleration.

Figure 6 is the durability analysis result according to the physical acceleration of the gel polymer reinforced membrane. For the commercial PE separator, since it is a porous support, gas was not blocked but permeated as it is. The GPERM took 132 s to lose 15% at 1 wt.% concentration of PEGMA oligomer while it recorded a remarkably high value of 2723 s when the concentration of PEGMA oligomer was 60 wt.%. This proves that PEGMA oligomer was well impregnated into the pores inside the PE separator, which is a porous support, and was gelatinized by the electron beam. On the other hand, for pure PEGMA gel, it only took 15 s to lose 15%. It confirmed that the gel itself has weak mechanical durability without a support. The physical acceleration analysis proved that the GPERM has significantly higher kinetic and mechanical durability than the existing gel polymer. It is considered that such gel formation will effectively prevent the short-circuit phenomenon between electrodes caused by dendrite formation.

**Figure 7:**
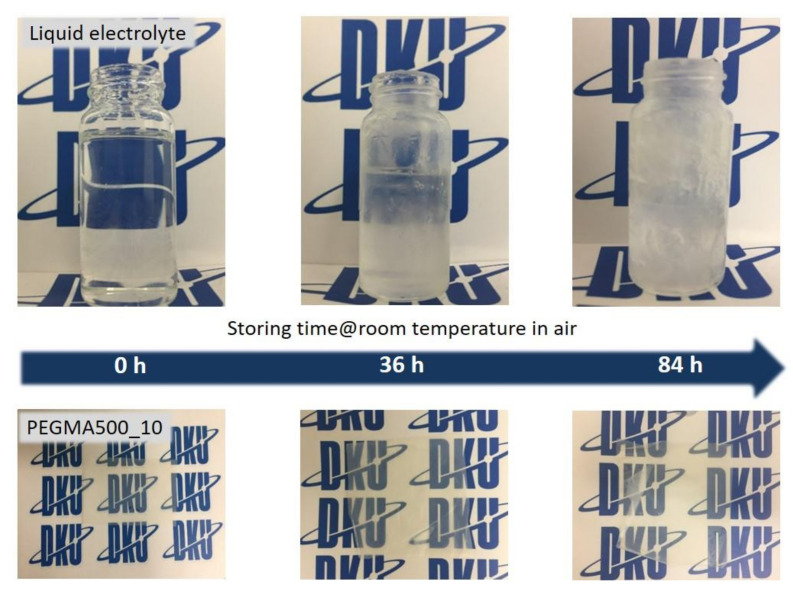
Digital camera images of the change in the state of the liquid electrolyte and GPERM.

**Figure 8:**
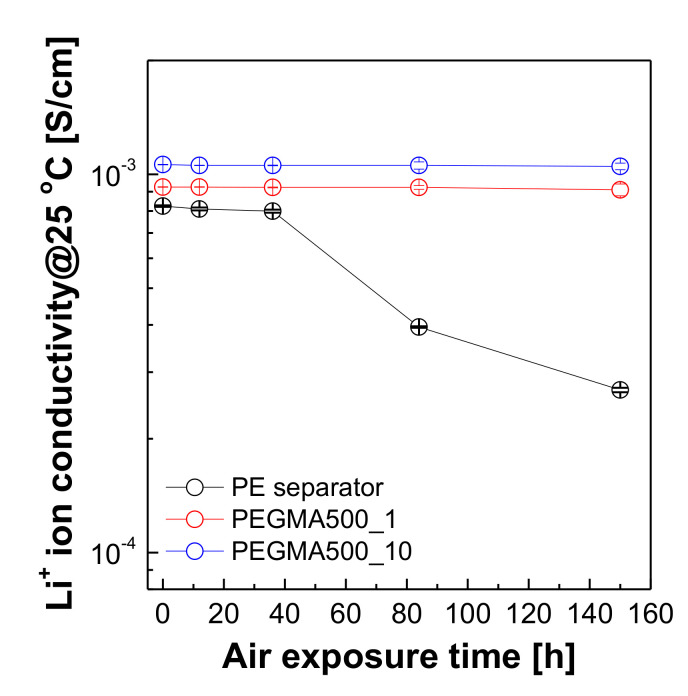
Lithium ion conductivity changes according to the different storage time.

Figure 7 is an image of the change in the state of the liquid electrolyte and GPERM based on the storage time. Since the liquid electrolyte is a carbonate-based electrolyte containing LiPF_6_, it was confirmed that the organic solvent volatilized and the lithium salt crystallized over time. On the other hand, the gel polymer electrolyte reinforced membrane hardly lost its initial transparency over time, and no lithium salt crystallized. This is predicted to be due to the physical maintenance of the carbonate-based electrolyte solution containing lithium salt due to the effect of the pouch structure inside the gel, which is described above, and the strong chemical bond of lithium ions with the oxy-ethylene unit formed inside the gel [45]. Figure 8 is data on the change in lithium ion conductivity according to the different storage times. As observed in Figure 8, the lithium ion conductivity decreases over time due to the precipitation of lithium salts. This difference was more pronounced in the liquid electrolyte than in the gel polymer electrolyte. This result is presumed to be due to the large impact of the absence of lithium, which is responsible for conductivity, due to the precipitation of lithium salt in the liquid electrolyte, and because of contamination by the reaction of lithium and moisture. In conclusion, this was an opportunity to prove that the gel polymer electrolyte reinforced membrane is more stable against exposure to external air and moisture than the liquid electrolyte.

**Figure 9:**
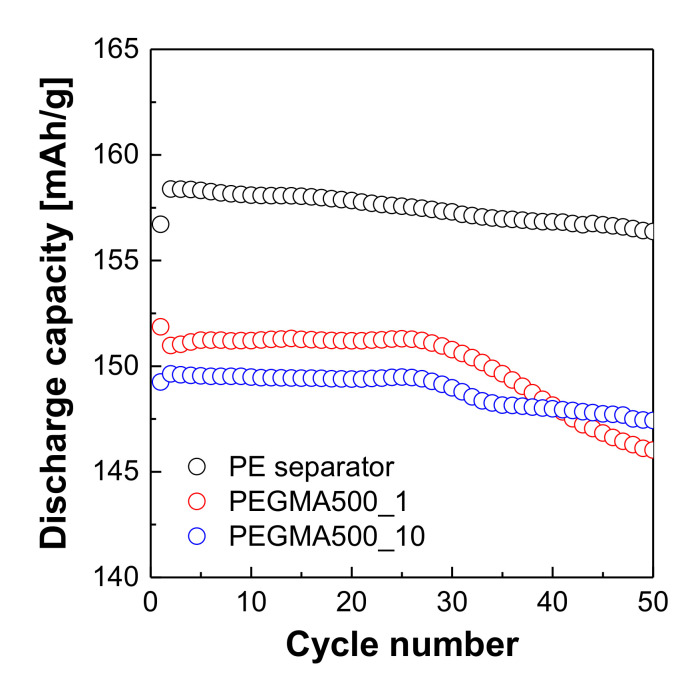
Cycle performance of the coin cells assemble by the commercial PE separator and GPERMs.

Figure 9 compares the cycle performance of coin cells with different separators and electrolytes. The coin cell with commercial PE separator and liquid electrolyte showed the highest initial discharge capacity and after 50 cycles at 1.0 C-rate, exhibited higher capacity retention (98.73%) than the other coin cells with the gel polymer electrolyte reinforced membrane. This appears to be a phenomenon caused by an uneven surface of the gel polymer that leads to an uneven interface with the electrodes. This phenomenon is more pronounced in PEGMA500_10 than in PEGMA500_1, in which the excessive growth of PEGMA oligomer in the thickness of PEGMA500_10 is thought to be negatively involved in the interfacial resistance with the electrode during charging and discharging. Even so, the coin cells with the gel polymer electrolyte reinforced membrane still show a relatively good capacity retention rate (>95%). It is thought that if PEGMA growth in thickness is controlled, the interface resistance with the electrode can be effectively reduced. In the meantime, the cycle performance of the coin cells will be improved.

## 4. Conclusions

In this study, a gel polymer electrolyte reinforced membrane was successfully fabricated with better lithium ion conductivity, thermal, and storage stability than the existing combination of liquid electrolyte and PE separator through the simultaneous irradiation cross-linking method using electron beam for the first time. Based on the porous PE separator, PEGMA was impregnated inside and cross-linked by an electron beam, and a pouch-shaped gel was created with uniformly distributed lithium ions, which was confirmed through SEM and TEM morphology analysis. It also proved that thermal stability, physical durability, and stability against moisture and air improved. When the gel polymer electrolyte reinforced membrane was introduced, the capacity retention rate was also relatively stable. Thus, it can be stated that the gel polymer electrolyte reinforced membrane is an innovative material for improving the properties of high-performance lithium-ion batteries.
